# Supplementary material for: Synthesis and Characterizations of Na4MnCr(PO4)3/rGO as NASICON-Type Cathode Materials for Sodium-ion Batteries
Source: Polymers (Basel). 2022 Sep 27;14(19):4046. doi: 10.3390/polym14194046 (PMC9570804; doi:10.3390/polym14194046)
Supplement: Supplementary file 1 [file polymers-14-04046-s001.zip › polymers-1929513-supplementary.pdf]

# Electronic Supporting Information

## Synthesis and Characterizations of $Na_4MnCr(PO_4)_3$/rGO as NASICON-type Cathode Materials for Sodium-ion Batteries

**Bing-Hsuan Hsu and Wei-Ren Liu***

*Department of Chemical Engineering, R&D Center for Membrane Technology, Center for Circular Economy, Chung Yuan Christian University, 200 Chung Pei Road, Chungli District, Taoyuan City 320, Taiwan*

**E-mail address: WRLiu1203@gmail.com;*

*Tel:+886 3-265-4140; Fax: +886 3-265-4199*

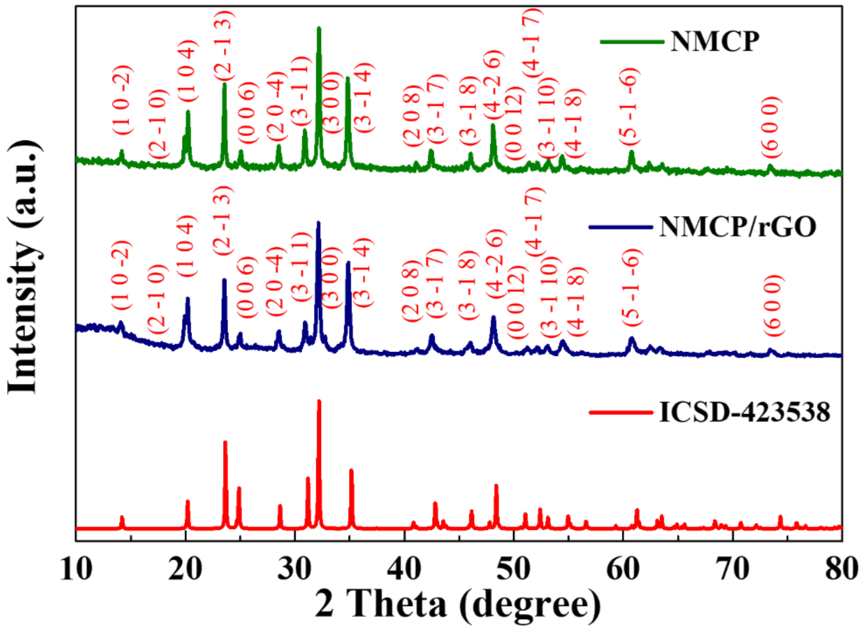


**Figure S1** X-ray diffraction (XRD) and corresponding Rietveld refinement results of NMCP.

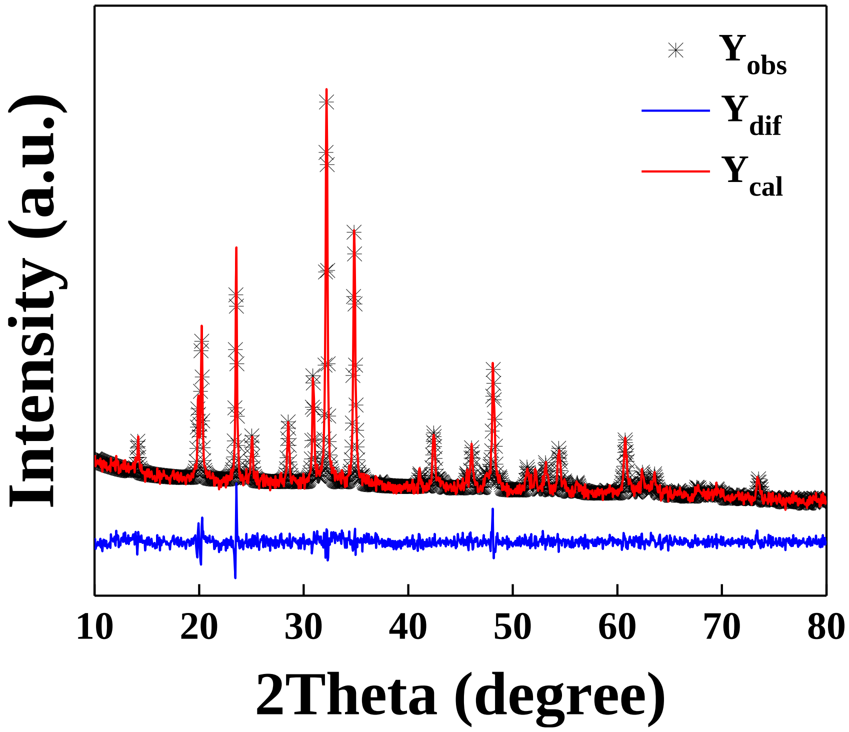


**Figure S2** X-ray diffraction (XRD) and corresponding Rietveld refinement results of NMCP.

**Table S1** Lattice parameters of pristine NMCP and as-synthesis NMCP/rGO.

| Samples | a (Å) | c (Å) | Cell volume (Å$^3$) | $R_{wp}$ |
|---|---|---|---|---|
| NMCP | 8.921 | 21.332 | 1470.245 | 6.631 |
| NMCP/rGO | 8.912 | 21.396 | 1471.612 | 10.821 |

**Table S2** Summary BET data of the NMCP and NMCP/rGO samples.

| Samples | Surface area ($m^2/g$) | Pore volume ($m^3/g$) | Pore size (nm) |
|---|---|---|---|
| NMCP | 34.202 | 0.0438 | 5.801 |
| NMCP/rGO | 12.631 | 0.0234 | 7.8081 |

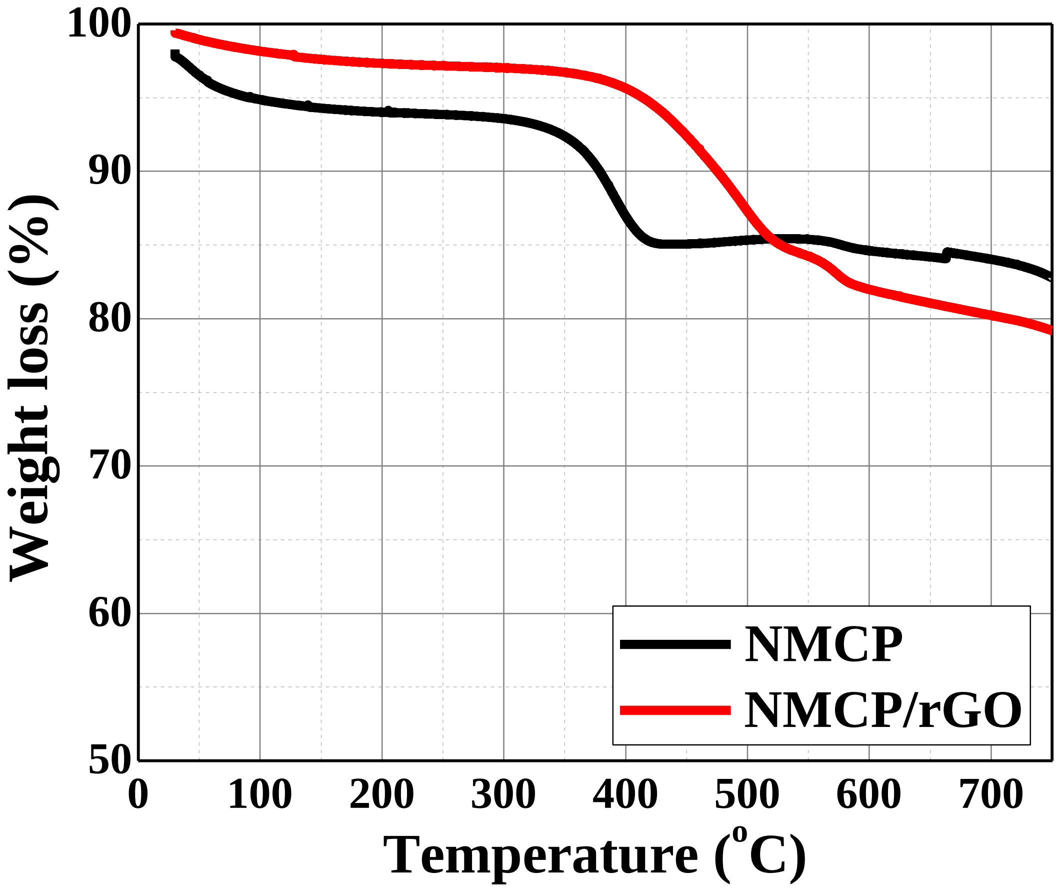


**Figure S3** TG analyses of pristine NMCP and NMCP/rGO composite under a heat rate of 5 ºC/min in air.

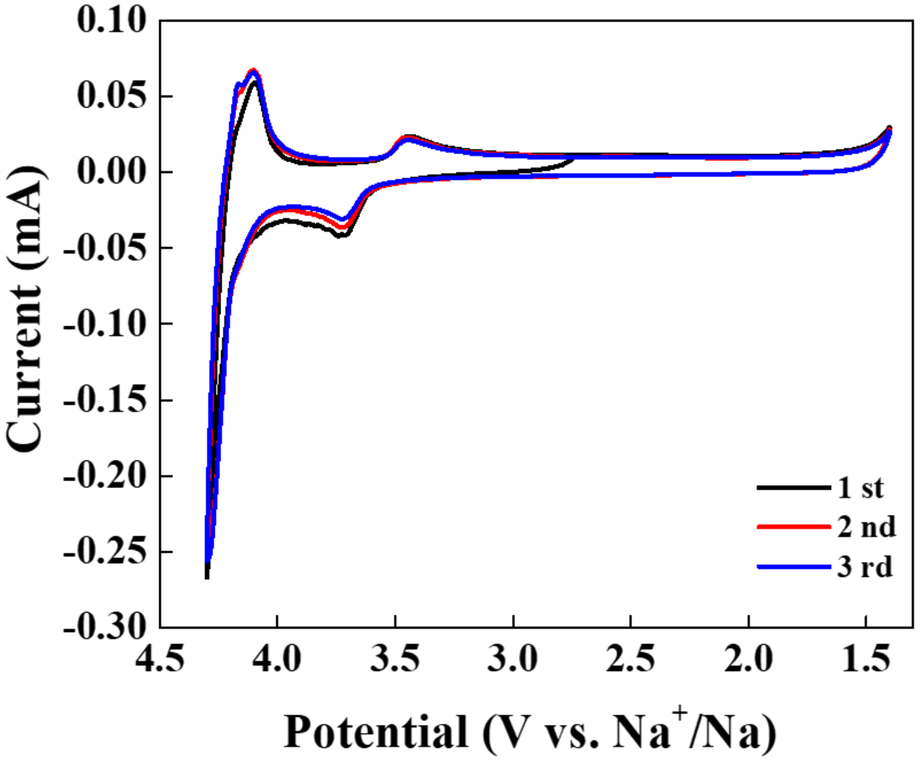


**Figure S4** Cyclic voltammetry profiles of pristine NMCP at 0.1 mV/s for three cycles.

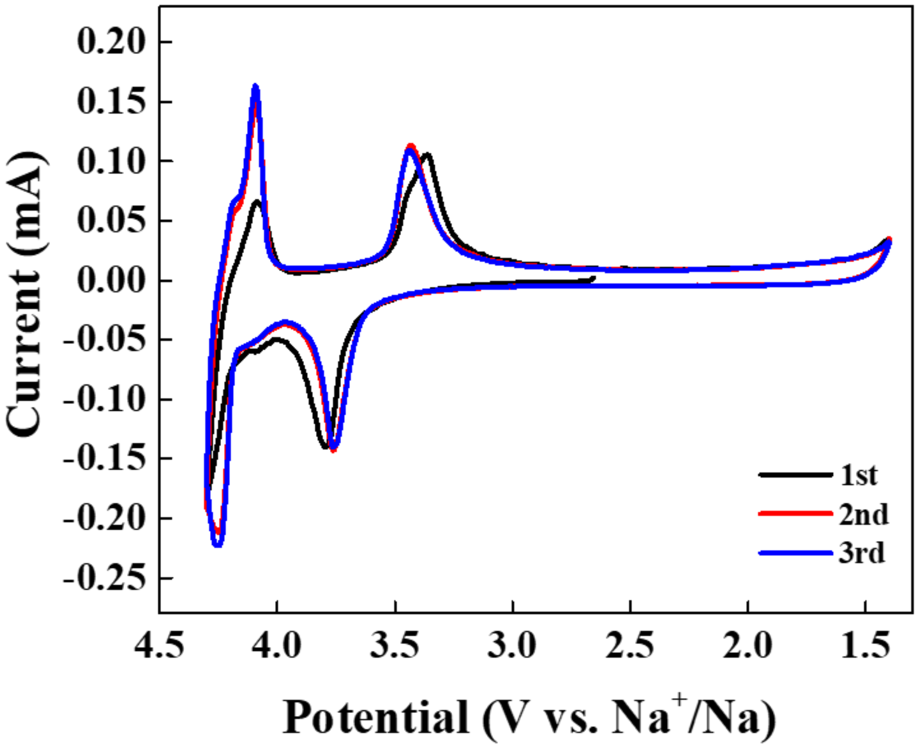


**Figure S5** Cyclic voltammetry profiles of as-synthesis NMCP/rGO at 0.1 mV/s for three cycles.

**Table S3** Electrode kinetic parameters obtained from equivalent circuit fitting of EIS for NMCP and NMCP/rGO samples.

| Material | $R_s$ (ohm) | $R_{ct}$ (ohm) | $R_{CEI}$ (ohm) | $\omega$ (ohm/$s^{1/2}$) |
|---|---|---|---|---|
| NMCP | 5.005 | 591.9 | 21.332 | 0.413 |
| NMCP/rGO | 3.995 | 102.7 | 32.37 | 30.19 |

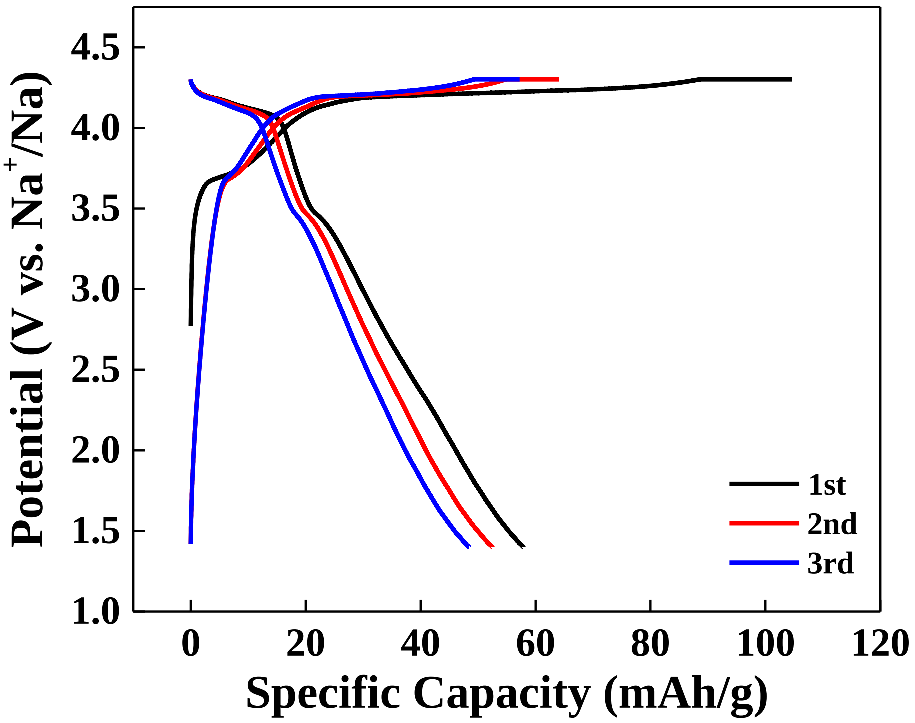


**Figure S6** Representative charge/discharge curves of NMCP at 0.01 A/g for three cycles.

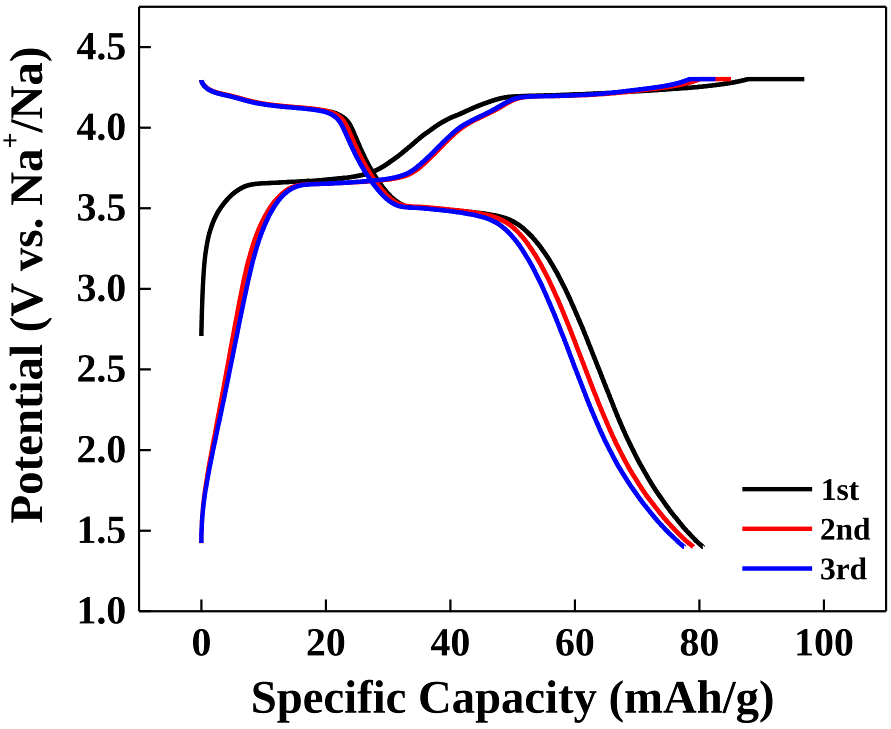


**Figure S7** Representative charge/discharge curves of NMCP/rGO at 0.01 A/g for three cycles.

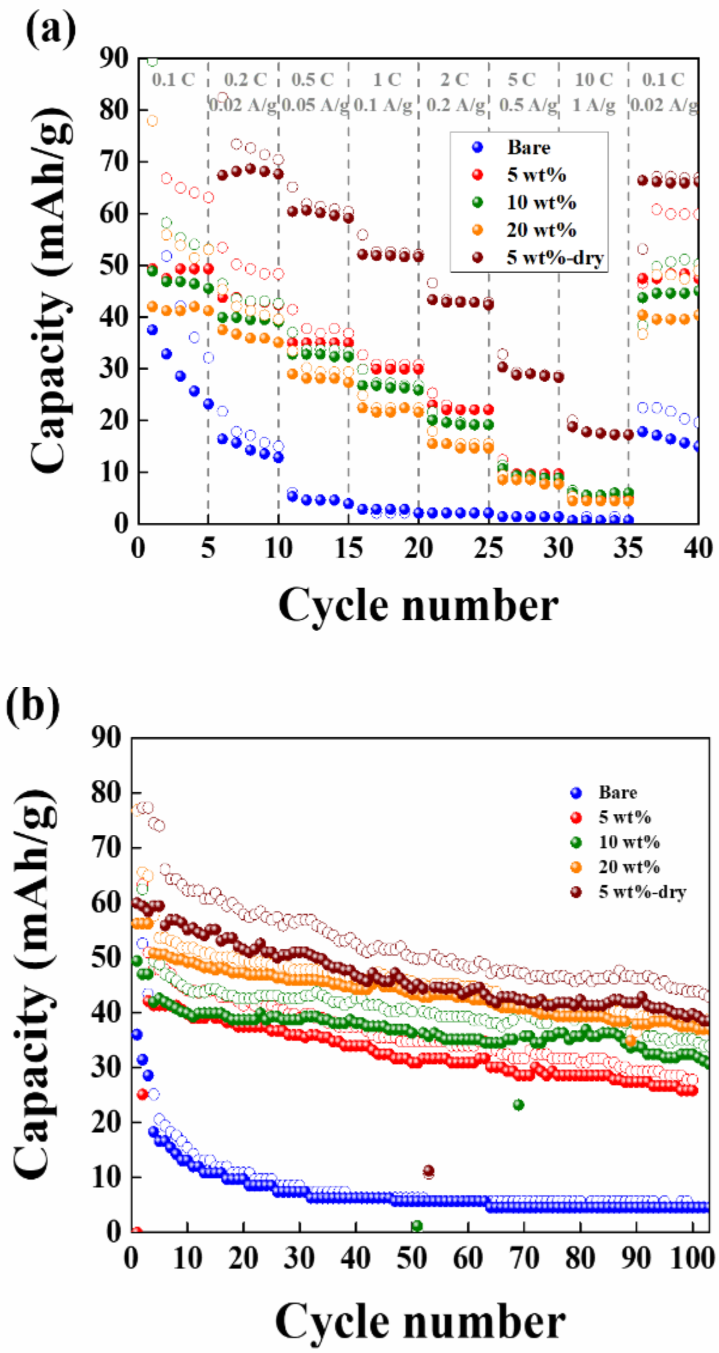


**Figure S8** Pristine NMCP and NMCP/rGO with different rGO content (a) C-rate (b) Cycle life.
